# The Bioactivity of *Glycyrrhizae Radix et Rhizoma Praeparata cum Melle* Carbon Dots: A Preliminary Study of Their Antiallergic Effect

**DOI:** 10.3390/cimb48050446

**Published:** 2026-04-24

**Authors:** Siqi Wang, Xiaohan Qu, Jinye Yuan, Jihang Zhang, Jiaxuan Zhang, Xinyu Huang, Jun Wang, Ziwen An, Yue Zhang, Hui Kong, Huihua Qu, Yan Zhao

**Affiliations:** 1 School of Traditional Chinese Medicine, Beijing University of Chinese Medicine, Beijing 100029, China; 2 School of Life Sciences, Beijing University of Chinese Medicine, Beijing 100029, China; 3 Center of Scientific Experiment, Beijing University of Chinese Medicine, Beijing 100029, China

**Keywords:** *Glycyrrhizae Radix et Rhizoma Praeparata cum Melle*, carbon dots, anti-allergic, MAPK

## Abstract

This study concurrently addressed the separation method for carbon dots derived from *Glycyrrhizae Radix et Rhizoma Praeparata cum Melle* (GRRPM) and the in vitro evaluation of their anti-allergic biological activity. *Glycyrrhizae Radix et Rhizoma Praeparata cum Melle* Carbon Dots (GRRPM-CDs) were prepared via decoction followed by dialysis, and their properties were characterized using High-Performance Liquid Chromatography (HPLC) and nanomaterial techniques. Anti-allergic activity was evaluated using a C48/80-induced RBL-2H3 mast cell degranulation model. Safety and efficacy were assessed using the CCK-8 assay, direct intervention, and drug-containing serum methods. The release of β-hexosaminidase (β-hex), histamine (HIS), interleukin-4 (IL-4), and tumor necrosis factor-α (TNF-α) was measured by ELISA, and key proteins in the MAPK signaling pathway were analyzed by Western blot. GRRPM-CDs inhibited mast cell degranulation and the release of allergic and inflammatory mediators in a dose-dependent manner. They also significantly downregulated the phosphorylation levels of the JNK, ERK, and p38 proteins in the MAPK signaling pathway. GRRPM-CDs exhibit significant anti-allergic activity, likely via suppression of the MAPK pathway. These findings provide new insights into the bioactive components of processed Glycyrrhiza and suggest potential avenues for developing novel therapies for allergic diseases.

## 1. Introduction

Allergic diseases are inflammatory conditions caused by dysregulation of the immune response to allergens [1], including allergic rhinitis, asthma, atopic dermatitis, food allergy, and drug allergy. In recent years, allergic diseases have affected approximately 10–30% of the global population, with preschool children being more commonly affected [2,3]. Numerous studies indicate that allergic rhinitis and allergic asthma can negatively affect patients’ health-related quality of life (HRQL) [4], and that atopic dermatitis is the leading cause of the economic burden among skin diseases [2]. Thus, research on the treatment of allergic diseases is particularly important.

Allergic diseases are closely associated with mast cells (MCs), which bind IgE with high affinity via the FcεRI (Fc epsilon RI) [5]. Mast cell degranulation releases large amounts of histamine and cytokines [6], critically influencing the acute onset of allergies, chronic inflammation, and tissue damage. Current treatments include conventional antihistamines, glucocorticoids, bronchodilators, leukotriene modulators, omalizumab (which blocks IgE binding to the FcεRI receptor) [7], tyrosine kinase inhibitors (which inhibit downstream signaling pathways) [8], and gene therapy [9]. However, most of these therapies are symptomatic, cannot provide a complete cure, and have significant side effects. Therefore, there is an urgent need to discover new drugs for clinical use.

*Glycyrrhizae Radix et Rhizoma Praeparata cum Melle* (GRRPM), a commonly used processed product of traditional Chinese medicine in clinical practice, has had its anti-allergic effect partially validated through both traditional use and modern research. This effect is often attributed to small-molecule bioactive compounds, such as glycyrrhizic acid and flavonoids.

Modern research has shown that glycyrrhizic acid, a major component of licorice, can inhibit mast cell degranulation and reduce the release of histamine and other inflammatory factors [10]; licorice extract can inhibit Th2 cell activation and suppress IgE production [11]. Thus, licorice exhibits clear anti-allergic effects; however, research on its bioactive components remains limited and has yet to fully elucidate its diverse pharmacological properties, such as anti-inflammatory [12], antioxidant [13], anti-tumor [14], and anti-diabetic activities [15], which demonstrate excellent performance in this area. Kong et al. [16] demonstrated that honey-processed licorice exhibits increased levels of active ingredients, such as glycyrrhetinic acid, thereby enhancing its immunomodulatory, anti-inflammatory, and hepatoprotective effects.

However, the material basis of the medicinal effect of GRRPM may be far more complex than what is currently known.

Our team’s preliminary research found that Chinese herbal medicines and their extracts can generate biologically active substances during high-temperature carbonization. Due to their unique nanoscale size and abundant surface functional groups, these substances are termed carbon dots (CDs) [17], which have broad applications in biomedicine [18] and have demonstrated efficacy in hemostasis [19], anti-inflammatory activity [20], and anti-ulcer effects [21]. Liu et al. [22] found that carbon dots formed via the specific carbonization of licorice constitute the material basis for its efficacy in treating gastric ulcers.

In recent years, CDs have emerged as a novel class of nanomaterials with significant potential in the biomedical field. At present, most research efforts have focused on carbon dots that are artificially synthesized or deliberately carbonized from plant materials. Conversely, there remains a lack of understanding regarding the presence of endogenous carbon dots in commercially available processed traditional Chinese medicines—and of their in situ generation during processing—including insights into their separation and identification, physicochemical properties, and biological activities. A crucial scientific question remains unresolved: Do these potential endogenous carbon dots constitute another important material basis for the anti-allergic effects of GRRPM? What is their mechanism of action? Therefore, the systematic isolation and characterization of these endogenous carbon dot components from GRRPM—and clarification of their role in anti-allergic activity—have become an urgent research gap that needs to be addressed.

This study aims to fill this gap and, for the first time, provides new material evidence and insights into the mechanism of the anti-allergic effect of the classic drug GRRPM.

Based on the above, this study employed a C48/80-induced degranulation model in RBL-2H3 cells and adopted two intervention strategies: direct drug administration and treatment with drug-containing serum. ELISA was used to detect the release levels of β-Hex, His, IL-4, and TNF-α; Western blotting was used to analyze the expression of key proteins in the MAPK signaling pathway (including JNK, ERK, and p38) and to investigate the anti-allergic activity and underlying mechanism of GRRPM-CDs. This study elucidates the material basis of GRRPM in treating allergic diseases from the perspective of carbon dots, providing new ideas for clinical treatment.

## 2. Materials and Methods

### 2.1. Materials

RBL-2H3 rat basophilic leukemia cells were purchased from Wuhan Zishan Biotechnology Co., Ltd. (product number: STCC30013, Wuhan, China). This cell line was archived in the Cell Bank of the Chinese Academy of Sciences (catalog number SCSP-518). *Glycyrrhizae Radix et Rhizoma Praeparata cum Melle* decoction pieces meeting the standards of the Chinese Pharmacopoeia were sourced from Beijing Qiancao Traditional Chinese Medicine Slices Co., Ltd. (batch number: 25021805, Beijing, China). The production process for *Glycyrrhizae Radix et Rhizoma Praeparata cum Melle* decoction pieces at this company is as follows: 100 kg of refined honey is diluted with 20 kg of boiling water. For every 100 kg of licorice slices, 25 kg of the diluted refined honey is used and mixed evenly. After moistening for 2 h, the mixture is placed in a hot pot and heated over a low flame at 80–120 °C. The drying temperature is set at 260 °C and maintained within the range of 240–260 °C. Each pot holds 50 kg of material and is stir-fried for 25 min until the surface turns deep yellow and no longer sticks to the hand. Then, it was removed, cooled, and made ready for use. The decoction of *Glycyrrhizae Radix et Rhizoma Praeparata cum Melle* was prepared in our laboratory. The dialysis membrane (1000 Da) was obtained from Beijing Ruida Henghui Technology Development Co., Ltd. (Beijing, China) C48/80 was sourced from Sigma-Aldrich (Shanghai) Trading Co., Ltd. (Shanghai, China). The Toluidine Blue staining solution was obtained from Beijing Solarbio Science & Technology Co., Ltd. (Beijing, China). The CCK-8 kit was purchased from Beijing BioRedGene Biotechnology Co., Ltd. (Beijing, China). Rat β-Hexosaminidase (β-Hex), Rat Histamine (HIS), Rat Interleukin 4 (IL-4), and Rat Tumor Necrosis Factor α (TNF-α) ELISA kits were all sourced from Shanghai Enzyme-linked Biotechnology Co., Ltd. (Shanghai, China). MEM basal medium, fetal bovine serum, penicillin–streptomycin mixture solution, and 0.25% trypsin digestion solution were all purchased from Wuhan Servicebio Technology Co., Ltd. (Wuhan, China). Rabbit monoclonal antibodies JNK (48 kD), p-JNK (37–46 kD), ERK1/2 (43 kD), p-ERK (38–43 kD), p38 MAPK (38–42 kD), and rabbit polyclonal antibody p-p38 MAPK (38–42 kD) were purchased from Wuhan Sanying Biotechnology Co., Ltd. (Wuhan, China). Rabbit polyclonal antibody β-actin and HRP-labeled goat anti-rabbit secondary antibody were sourced from Proteintech Group, Rosemont, IL, USA. All experiments were conducted using deionized water.

### 2.2. Cell Culture

RBL-2H3 cells were cultured in MEM complete medium (89% MEM medium, 10% fetal bovine serum, 1% penicillin–streptomycin mixture (100×)) under conditions of 5% CO_2_ and 37 °C.

### 2.3. Preparation of GRRPM-CDs

GRRPM-CDs were isolated from commercially accessible GRRPM decoction pieces. After crushing, 200 g of GRRPM powder was weighed, wrapped in non-woven gauze, placed in a beaker, soaked in 2 L of deionized water for 15 min, and heated twice in a 100 °C constant-temperature water bath. The mixture was filtered twice to remove residues, and the filtrates were combined and concentrated using a rotary evaporator to obtain the original solution of GRRPM. This solution was placed in a 1000 Da dialysis bag and dialyzed against deionized water for 7 days, with frequent water changes to ensure complete removal of low-molecular-weight substances until the external solution became colorless and transparent. The dialyzed solution was centrifuged at 8000 rpm for 20 min to remove precipitates formed by aggregation during dialysis. The supernatant was concentrated and adjusted to a concentration of 1 g/mL (based on the weight of GRRPM decoction pieces), yielding the GRRPM-CD solution, which was then stored at 4 °C. The high-concentration GRRPM-CD solution was obtained by evaporating and concentrating the 1 g/mL GRRPM-CD solution to a concentration five times its original value, specifically 5 g/mL.

### 2.4. HPLC Analysis of GRRPM Solution and GRRPM-CDs

An Agilent 1260 Infinity high-performance liquid chromatograph (Santa Clara, CA, USA) equipped with a ZORBAX-C18 column (4.6 mm × 250 mm × 5 μm) was used to detect small-molecule compounds in the original *Glycyrrhizae Radix et Rhizoma Praeparata cum Melle* solution and the GRRPM-CD solution. All the test samples were prepared by diluting the standard solution with a relative concentration of 1 g/mL by a factor of 500. These solutions were prepared as described previously using deionized water. Mobile phase A was 0.1% formic acid, and mobile phase B was acetonitrile, with a flow rate of 1.0 mL/min; the injection volume was 10 μL. The elution gradient was as follows [23]: 0–7 min, 10–22% B; 7–12 min, 22–28% B; 12–15 min, 28% B; 15–18 min, 28–29% B; 18–20 min, 29–30% B; 20–23 min, 30–31% B; 23–31 min, 31–46% B; 31–34 min, 46–50% B; 34–44 min, 50–57% B; 44–46 min, 57–60% B; 46–50 min, 60% B. The detection wavelength is 237 nm.

### 2.5. Determination of the Yield of GRRPM-CDs

To quantify the separation efficiency of GRRPM-CDs, the mass yield was determined by freeze-drying. After dialysis purification, the GRRPM-CD solution was transferred into a pre-weighed freeze-drying vial. The sample was then placed in a freeze dryer and lyophilized at a temperature below −50 °C and a vacuum pressure lower than 10 Pa for 24–48 h until a dry, free-flowing powder was obtained. The vial containing the dried product was accurately reweighed. The mass yield of GRRPM-CDs was calculated based on the mass of the initial raw materials.

### 2.6. Characterization Methods of GRRPM-CDs

The physical morphology and structural characterization of GRRPM-CDs were observed using a low-resolution transmission electron microscope (TEM, Tecnai G220, FEI Company, Hillsboro, OR, USA) and a high-resolution transmission electron microscope (HRTEM, JEM-1230, JEOL Ltd., Tokyo, Japan). The crystal structure was analyzed using an X-ray diffractometer (XRD, D8 DISCOVER, Bruker Corporation, Billerica, MA, USA). Functional groups and optical properties were analyzed using a Fourier-transform infrared spectrometer (FTIR, Nicolet iS10, Thermo Fisher Scientific Inc., Waltham, MA, USA), an X-ray photoelectron spectrometer (XPS, ESCALAB 250Xi, Thermo Fisher Scientific Inc., Waltham, MA, USA), an ultraviolet–visible spectrophotometer (UV-Vis, Cecil Instruments Ltd., Cambridge, UK), and a fluorescence spectrophotometer (F-4500, Thermo Fisher Scientific Inc., Waltham, MA, USA). All the test samples were obtained from the standardized 1 g/mL GRRPM-CD solution prepared in the previous section.

### 2.7. Cell Viability Assay

Experiments were performed strictly following the instructions for the CCK-8 kit. RBL-2H3 cells were adjusted to a density of 2 × 10^5^ cells/mL using MEM complete medium, inoculated into 96-well plates at 100 μL/well, and incubated for 24 h. Test samples were added after washing twice with PBS. After treatment, 100 μL of complete medium containing 10% CCK-8 reagent was added to each well, and the plates were incubated for 1 h. Absorbance (OD) at 450 nm was measured using a microplate reader (BioTek Instruments, Inc., Winooski, VT, USA).

Cell survival rate was calculated as [(As − Ab)/(Ac − Ab)] × 100%, where As = absorbance of the experimental group, Ab = absorbance of the blank group, and Ac = absorbance of the control group. This allowed analysis of the cell survival percentage under specific conditions to evaluate the impact of test conditions on cell viability and to select optimal experimental conditions.

### 2.8. Determination of C48/80 Concentration

#### 2.8.1. CCK-8 and Toluidine Blue Staining

A 0.4 mg quantity of C48/80 powder was dissolved in MEM complete medium to prepare an 80 μg/mL solution, sterilized twice using a 0.22 μm syringe filter, then serially diluted to 40, 20, 10, 5, and 2.5 μg/mL, and stored at 4 °C. The safe concentration range of C48/80 was determined using the CCK-8 assay and toluidine blue staining.

#### 2.8.2. β-Hexosaminidase Release Assay

RBL-2H3 cells were stimulated with 2.5, 5, 10, and 20 μg/mL C48/80 for 30 min, and β-hexosaminidase concentration was measured using an ELISA kit according to the manufacturer’s instructions. The degree of cell degranulation induced by different concentrations of C48/80 was determined based on β-hexosaminidase release. The optimal concentration (20 μg/mL) was then used to stimulate cells for 5, 10, 15, 20, 25, and 30 min, and the assay was repeated to determine the optimal stimulation time.

### 2.9. Safety Evaluation of GRRPM-CDs

Prepared GRRPM-CD solution was evaporated, weighed, and reconstituted in MEM complete medium to concentrations of 3000, 1500, 750, 375, 187.5, 93.75, 46.87, and 23.45 μg/mL, sterilized twice using a 0.22 μm filter, and stored at 4 °C. The safety of GRRPM-CDs was evaluated using the CCK-8 method.

### 2.10. Preparation and Concentration Determination of Drug-Containing Serum

#### 2.10.1. Preparation of Drug-Containing Serum

Six SPF-grade male SD rats (6–7 weeks old, 200 ± 10 g) were purchased from Beijing Vital River Laboratory Animal Technology Co., Ltd. (Beijing, China). The study was approved by the Ethics Committee for Animal Experimentation of Beijing University of Traditional Chinese Medicine (BUCM-2024070804-3033) and complied with the Guidelines for the Care and Use of Laboratory Animals. Rats were housed under standard conditions (22 ± 1 °C, 50–65% humidity, 12 h light/dark cycle) with ad libitum access to food and water. After 3 days of acclimatization, the rats were randomly divided into a drug administration group (*n* = 4, gavaged with 3 mL of a 5 g/mL high-concentration GRRPM-CD solution) and a control group (*n* = 2, gavaged with an equal volume of saline). Two hours after administration, blood was collected from the abdominal aorta, placed in non-anticoagulant tubes for 2 h, and centrifuged at 3000 rpm for 15 min at 4 °C to obtain serum. The serum was inactivated in a 56 °C water bath for 30 min, filtered through a 0.22 μm membrane for sterilization, and stored at −20 °C.

#### 2.10.2. Determination of Drug-Containing Serum Concentration

Experiments included three groups: the control group (no addition), the drug-containing serum group (5%, 10%, 15%, or 20% drug-containing serum in MEM complete medium), and the blank serum group (5%, 10%, 15%, or 20% blank serum in MEM complete medium). The effect of serum concentration on RBL-2H3 cell viability was evaluated by determining cell survival rates at 24 and 48 h using the CCK-8 assay.

### 2.11. Evaluation of Anti-Allergic Effects

The release of β-Hex, HIS, IL-4, and TNF-α was detected using ELISA kits.

#### 2.11.1. Direct Drug Interventions

RBL-2H3 cells in 96-well plates were randomly assigned to control, model (C48/80), and high-dose (7.5 μg/mL), medium-dose (3.75 μg/mL), and low-dose (1.875 μg/mL) GRRPM-CD groups, with four replicate wells per group.

#### 2.11.2. Drug-Containing Serum Intervention

RBL-2H3 cells in 6-well plates were randomly divided into control, model (C48/80), and drug-containing serum groups (treated with 10% drug-containing serum in MEM complete medium). The control and model groups received the same volume of blank serum medium.

### 2.12. Western Blot Detection of MAPK Pathway Protein Expression

RBL-2H3 cells in 6-well plates were randomly divided into control, model (C48/80), and drug-containing serum (10% drug-containing serum in MEM complete medium) groups. The control and model groups received the same volume of blank serum medium.

Logarithmic-phase RBL-2H3 cells were collected, lysed, and centrifuged to obtain protein samples, which were denatured after concentration determination using the BCA assay. Samples were subjected to SDS-PAGE, transferred to PVDF membranes, blocked, and incubated sequentially with primary antibodies and HRP-labeled secondary antibodies. Bands were visualized using an ECL chemiluminescence kit, and grayscale values were analyzed using Image-Pro Plus 6.0 to evaluate protein expression in the MAPK pathway.

### 2.13. Statistical Analysis

The number of replicates ranges from 4 to 6. Data were analyzed using IBM SPSS 25.0. Measurement data are expressed as mean ± standard deviation ($\bar{x}$ ± s). Normality and homogeneity of variance tests were performed first. For normally distributed data with homogeneous variance, one-way ANOVA was used, with intergroup comparisons conducted using the LSD test. Differences were considered statistically significant at *p* < 0.05.

## 3. Results

### 3.1. HPLC Analysis of GRRPM and GRRPM-CDs

**Figure 1:**
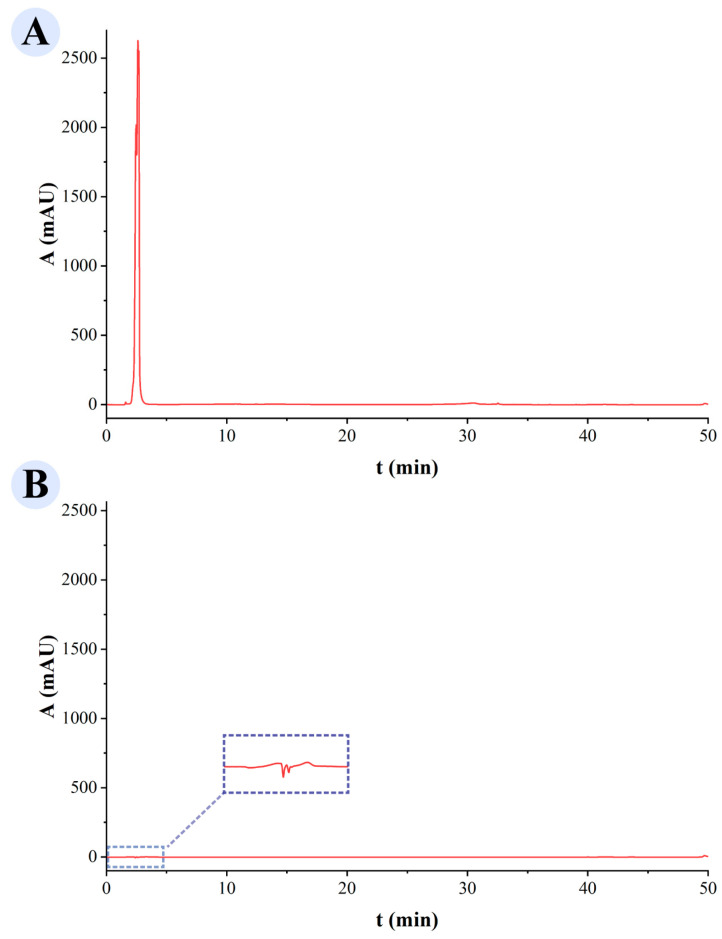
HPLC profiles: (**A**) *Glycyrrhizae Radix et Rhizoma Praeparata cum Mell*; (**B**) GRRPM-CDs.

The HPLC results of the GRRPM solution prior to dialysis (Figure 1A) exhibit an anomalously sharp and high-intensity peak at a retention time of approximately 3–5 min, with a response value exceeding 2500 mAU. This outcome suggests that the GRRPM solution is rich in small-molecule compounds. After dialysis, the GRRPM-CD solution (Figure 1B) shows that the aforementioned strong absorption peak has nearly vanished within the identical retention time range. Meanwhile, no other notable chromatographic peaks were detected across the entire scanning range. This indicates that the dialysis purification process employed in this study is capable of effectively separating GRRPM-CDs from the vast majority of well-known small-molecule compounds (e.g., liquiritin) present in the raw materials. This ensures that subsequent spectroscopic characterizations (e.g., FTIR, XPS) and biological activity evaluations primarily focus on the GRRPM-CDs as the nanocomponent, rather than on residual traditional small-molecule components.

### 3.2. The Yield of GRRPM-CDs

To assess the extraction efficiency of GRRPM-CDs, we determined their yield. After freeze-drying, a brownish, hygroscopic solid powder was obtained. Based on the mass of the starting materials, the yield of GRRPM-CDs was 0.0188% (*w*/*w*).

### 3.3. Characterization of GRRPM-CDs

**Figure 2:**
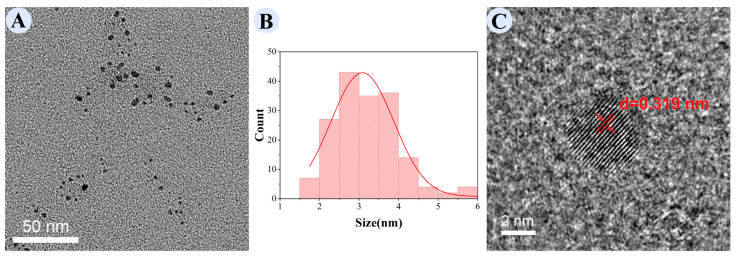
Transmission electron microscopy of GRRPM-CDs: (**A**) TEM; (**B**) particle size distribution; (**C**) enlarged lattice image; the arrow indicates the lattice fringes.

TEM (Figure 2A,B) showed that GRRPM-CDs had a regular near-spherical nanostructure with uniform dispersion and a particle size range of 1.0–6.0 nm, peaking at approximately 3 nm (consistent with a normal distribution). HRTEM (Figure 2C) revealed clear lattice fringes on the nanoparticle surface with a lattice spacing of 0.319 nm.

**Figure 3:**
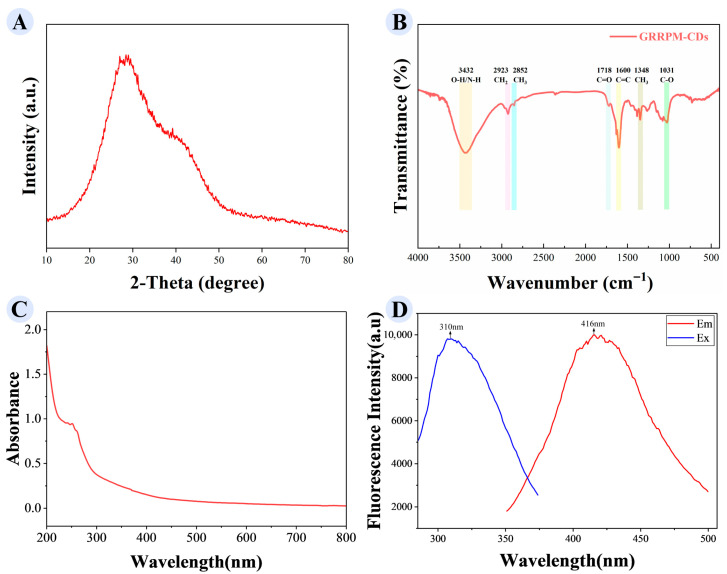
Spectral characterization of GRRPM-CDs: (**A**) XRD; (**B**) FTIR spectra; (**C**) UV-Vis spectra; (**D**) fluorescence spectra.

XRD analysis (Figure 3A) showed a diffraction peak at 2θ = 27.354°, corresponding to a lattice spacing of ~0.325 nm (consistent with HRTEM observations). FTIR spectroscopy (Figure 3B) identified functional groups in GRRPM-CDs: absorption peaks at 3432 cm^−1^ (O-H or N-H stretching), 2923 cm^−1^ and 2852 cm^−1^ (C-H stretching of CH_2_ and CH_3_, respectively), 1718 cm^−1^ (C=O), 1600 cm^−1^ (C=C stretching), 1348 cm^−1^ (methyl bending), and 1031 cm^−1^ (C-O stretching). These indicate the presence of reactive groups (amino, hydroxyl, carboxyl), which may contribute to their pharmacological effects.

Optical property analysis (Figure 3C) showed a weak UV absorption peak at 251 nm, suggesting electronic transitions in unsaturated or aromatic groups. Fluorescence spectra (Figure 3D) showed maximum excitation at 310 nm and emission at 416 nm.

**Figure 4:**
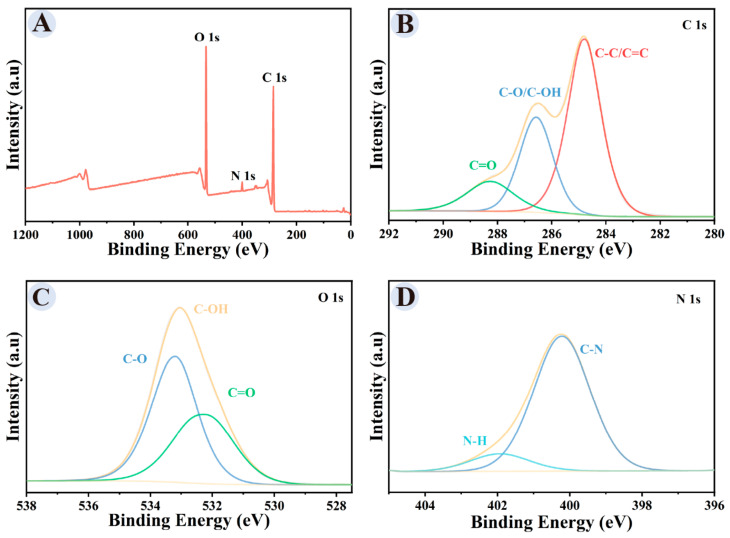
XPS spectra of GRRPM-CDs: (**A**) full scan; (**B**) C 1s; (**C**) O 1s; (**D**) N 1s.

XPS (Figure 4) showed that GRRPM-CDs mainly contained C (69.57%), O (27.59%), and N (2.84%). The C 1s spectrum exhibited peaks at 284.7 eV (C–C/C=C), 286.4 eV (C–OH/C–O), and 288.3 eV (C=O). The O 1s spectrum exhibited peaks at 532.2 eV (C=O), 533.1 eV (C–OH), and 533.2 eV (C–O). The N 1s spectrum exhibited peaks at 400.2 eV (C–N) and 401.9 eV (N–H).

### 3.4. Optimal Conditions for RBL-2H3 Degranulation Modeling

**Figure 5:**
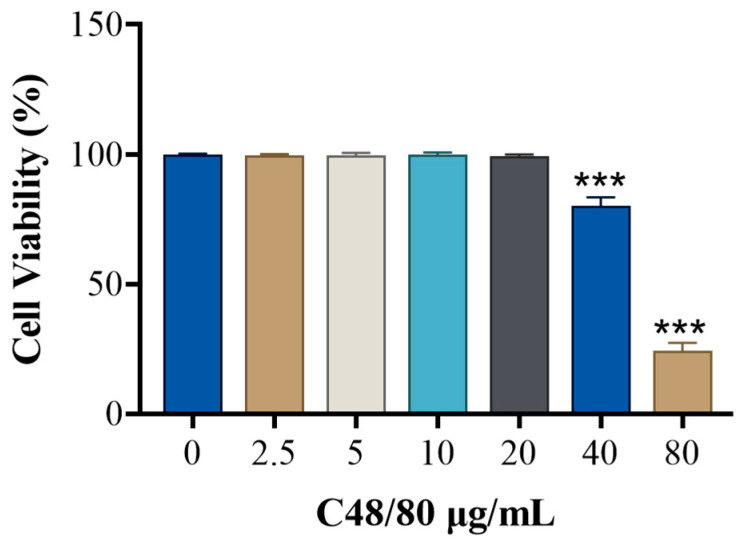
Effect of C48/80 concentration on RBL-2H3 viability. Each group *n* = 6. Compared with control, *** *p* < 0.001.

**Figure 6:**
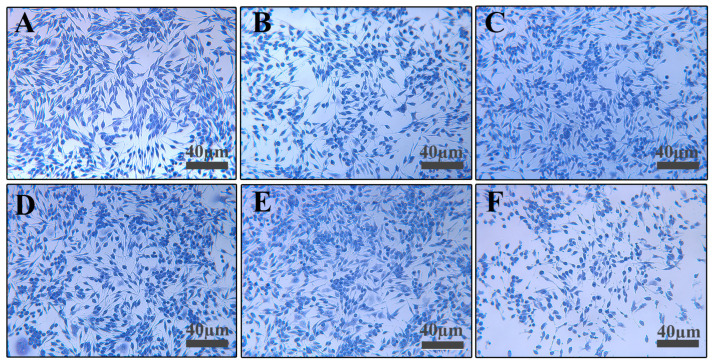
Effect of C48/80 on RBL-2H3 morphology (100×): (**A**) 0 μg/mL; (**B**) 2.5 μg/mL; (**C**) 5 μg/mL; (**D**) 10 μg/mL; (**E**) 20 μg/mL; (**F**) 40 μg/mL.

CCK-8 assay (Figure 5) showed no significant cytotoxicity when RBL-2H3 cells were stimulated with 2.5–20 μg/mL C48/80 for 30 min. Toluidine blue staining (Figure 6) showed morphological changes (rounding, swelling, loss of luster) at 40 μg/mL C48/80; within the concentration range of 40–80 µg/mL, cell viability decreased in a dose-dependent manner with the increase in the concentration of C48/80. Therefore, concentrations below 20 μg/mL were considered safe.

**Figure 7:**
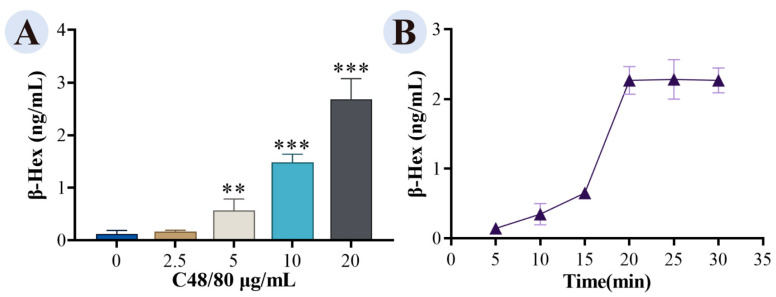
β-Hex content in cell supernatants: (**A**) effect of C48/80 concentration; (**B**) effect of stimulation time with 20 μg/mL C48/80. Each group *n* = 6. Compared with blank, *** *p* < 0.001, ** *p* < 0.01.

β-Hex release increased with C48/80 concentration (2.5–20 μg/mL; Figure 7A), with 20 μg/mL inducing maximum degranulation without cytotoxicity. Time-course analysis (Figure 7B) showed that β-Hex release peaked at 20 min with 20 μg/mL C48/80 and remained stable thereafter. Thus, the optimal modeling condition was 20 μg/mL C48/80 for 20 min.

### 3.5. Safety of GRRPM-CDs

#### 3.5.1. Direct Drug Delivery: Safety Evaluation

**Figure 8:**
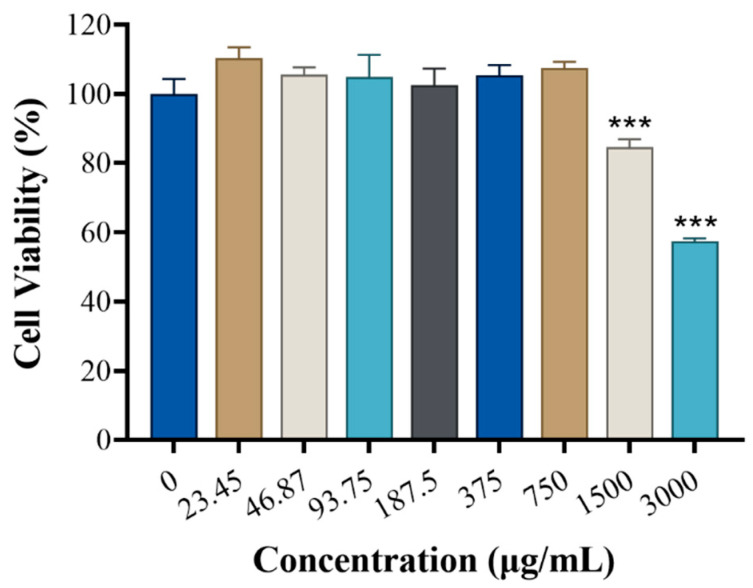
Cytotoxicity of GRRPM-CDs. Each group *n* = 4. Compared with blank, *** *p* < 0.001.

CCK-8 assay (Figure 8) showed no reduction in RBL-2H3 cell viability at GRRPM-CDs concentrations ≤750 μg/mL; viability was inhibited at 1500 μg/mL. Based on safety considerations and proximity to in vivo exposure levels, the highest concentration for subsequent experiments was set at 7.5 μg/mL (below the IC_10_), with gradient doses administered.

#### 3.5.2. Drug-Containing Serums

Table 1 shows that 15% and 20% blank or drug-containing serum significantly reduced the 24 h and 48 h RBL-2H3 cell survival, indicating that high serum concentrations (>15%) inhibited viability. Table 2 shows that C48/80 significantly increased β-Hex release (*p* < 0.001), confirming the establishment of the model. While 5% and 10% blank serum had no effect on β-Hex release, 5% and 10% drug-containing serum significantly reduced it (*p* < 0.001), with 10% drug-containing serum showing the strongest inhibitory effect.

### 3.6. Anti-Allergic Effects

C48/80-induced RBL-2H3 degranulation disrupts intracellular homeostasis, triggering inflammatory responses and immune disorders associated with mast cell activation and mediator release. This study evaluated the effects of GRRPM-CDs and serum containing GRRPM-CDs on the release of β-Hex, His, IL-4, and TNF-α, combined with morphological observations, to assess their impact on degranulation-associated inflammation.

#### 3.6.1. Direct Drug Delivery

**Figure 9:**
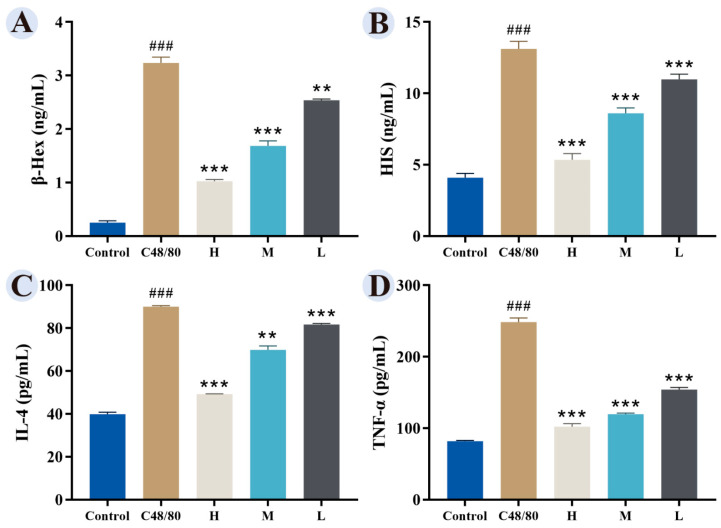
Effects of GRRPM-CDs on β-Hex and inflammatory factor release in RBL-2H3 cells. (**A**) β-Hex release. (**B**) His release. (**C**) IL-4 release. (**D**) TNF-α release. Groups: Control; C48/80 (model); H (high dose); M (medium dose); L (low dose). Compared with blank, ### *p* < 0.001; Compared with the model group, *** *p* < 0.001, ** *p* < 0.01.

Compared with the control group, the model group showed significantly elevated levels of β-Hex, His, IL-4, and TNF-α. GRRPM-CDs at high (7.5 μg/mL), medium (3.75 μg/mL), and low (1.875 μg/mL) doses reduced these levels, with the high-dose group showing the strongest effect, indicating a dose-dependent inhibitory effect (Figure 9).

**Figure 10:**
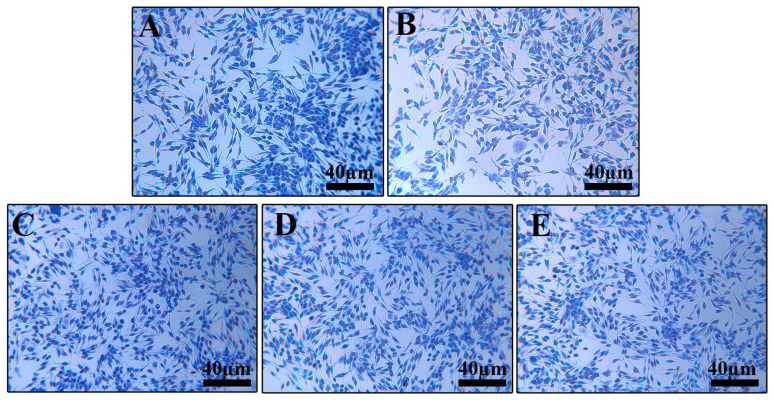
Effect of GRRPM-CDs on toluidine blue staining of RBL-2H3 cells (200×): (**A**) Control; (**B**) C48/80; (**C**) high dose; (**D**) medium dose; (**E**) low dose.

Toluidine blue staining (Figure 10) showed clear, typically spindle-shaped cells with uniform cytoplasmic staining in the control group. The model group exhibited granule release and reduced staining intensity. GRRPM-CD intervention improved cell morphology in a dose-dependent manner.

#### 3.6.2. Delivery of Drug-Containing Serums

**Figure 11:**
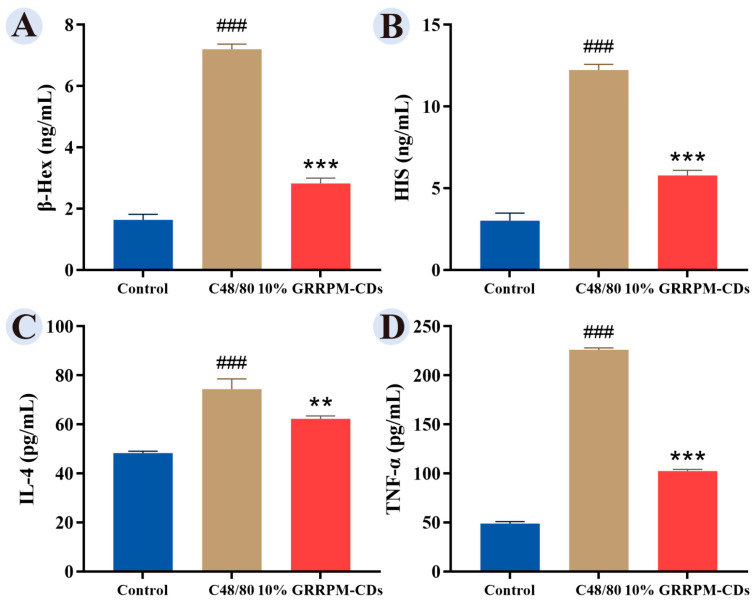
Effect of 10% GRRPM-CDs-containing serum on cytokine expression in RBL-2H3 cells, including β-HEX (**A**), HIS (**B**), IL-4 (**C**), and TNF-α (**D**). Compared with the blank group, ### *p* < 0.001; Compared with the model group, *** *p* < 0.001, ** *p* < 0.01.

The model group showed significantly elevated β-HEX, HIS, IL-4, and TNF-α levels compared with the control group, while serum containing 10% GRRPM-CDs reduced these levels (Figure 11).

**Figure 12:**
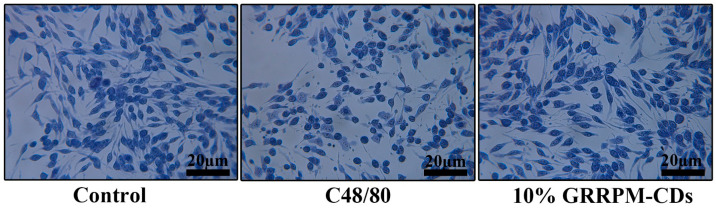
Effect of GRRPM-CDs-containing serum on toluidine blue staining of RBL-2H3 cells (400×): Control; C48/80; 10% GRRPM-CDs.

Toluidine blue staining (Figure 12) showed normal morphology with few intracellular granules in the control group; the model group exhibited disorganized morphology and granule exudation; the 10% drug-containing serum group preserved cell membrane integrity with minimal granule release.

### 3.7. Effects of GRRPM-CDs on MAPK Pathway-Related Protein Expression

**Figure 13:**
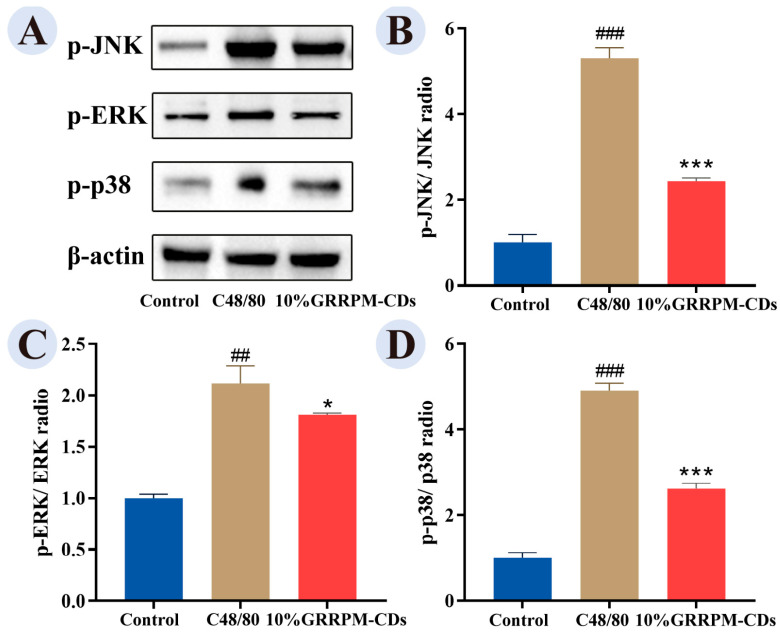
Immunoblot analysis of MAPK pathway proteins: (**A**) WB bands; (**B**) p-JNK/β-actin; (**C**) p-ERK/β-actin; (**D**) p-p38/β-actin. Compared with the control group, ### *p* < 0.001, ## *p* < 0.01; Compared with the model group, *** *p* < 0.001, * *p* < 0.05.

The MAPK signaling pathway regulates mast cell activation and inflammatory mediator release [24]. Compared with the control group, the model group showed increased expression of p-ERK, p-JNK, and p-p38. These levels were reduced in the drug-containing serum group (Figure 13), suggesting that GRRPM-CDs may inhibit mast cell activation by downregulating MAPK pathway proteins.

## 4. Discussion

Carbon dots derived from traditional Chinese medicine are emerging nanomaterials that integrate the characteristics of traditional Chinese medicine with those of nanomaterials, featuring high specific surface areas and abundant surface-active sites [25]. They can bind to cell surface receptors or signaling molecules even at low concentrations, demonstrating broad biomedical applications owing to their unique physicochemical and biological properties. Previous studies have demonstrated their advantages in anti-allergy [26], anti-inflammation [27], and hemostasis [19], with strong targeting, low toxicity, and versatility.

Some recent studies have indicated that glycyrrhizin blocks inflammatory signaling by inhibiting NF-κB activation in histamine-induced human nasal epithelial cells [28], and that glycyrrhetinic acid reduces allergic rhinitis inflammation in mice by enhancing antioxidant capacity and correcting the Th1/Th2 imbalance. Clinical trials show that licorice extract nasal irrigation is more effective than saline for allergic rhinitis [29].

Based on the above theoretical foundation, this study was conducted to investigate the therapeutic mechanism of *Glycyrrhizae Radix et Rhizoma Praeparata cum Melle* as an important component underlying the efficacy of GRRPM-CDs in treating allergic diseases. The anti-allergic properties of small-molecule constituents, such as glycyrrhizic acid, have been comprehensively validated in previous research. This study has found that GRRPM-CDs are a nanoscale entity distinct from small molecules such as glycyrrhizic acid and glycyrrhizin. Despite differences in chemical structure, both may exert similar or complementary biological effects—namely, inhibition of mast cell degranulation and modulation of the MAPK signaling pathway. This indicates that the anti-allergic effect of processed licorice might be the outcome of the combined action of multiple active components. Future research can conduct a more in-depth comparison of the differences in efficacy and mechanism between CDs and monomer components such as glycyrrhizic acid.

The pathogenesis of allergic diseases is complex, and immune abnormalities are one of the major causative factors, in which imbalances of cytokines and chemokines play a key role in disease progression. When an allergen enters the body for the first time, the specific IgE antibody produced binds with high affinity to the FcεRI receptor on the surface of mast cells [30]. Mast cell activation can be triggered when allergens re-invade and bind to IgE. Activated mast cells rapidly degranulate and release mediators such as histamine, proteases, leukotrienes, and prostaglandins [31], causing allergic symptoms. Meanwhile, the activated mast cells can also synthesize and secrete various cytokines and chemokines, such as IL-4, IL-5, IL-13, TNF-α [32], contributing to the development of chronic allergic inflammation. Therefore, maintaining mast cell stability is an important anti-allergic mechanism.

In this study, we selected β-Hex, histamine, IL-4, and TNF-α as indicators to assess the allergic state, covering a comprehensive cascade from effector cells (mast cells) to inflammatory mediators (histamine), adaptive immune factors (IL-4), and innate immune factors (TNF-α).

The results indicated that indicators related to cell inflammation were significantly elevated in the model group. However, following the intervention with GRRPM-CDs, both direct and serum-containing drug delivery methods demonstrated a reduction in the release of β-Hex and histamine, as well as a decrease in the levels of IL-4 and TNF-α.

Therefore, GRRPM-CDs play a role in the initiation (β-Hex) and effector phase (histamine) of allergic reactions, and deeply regulate immune cell differentiation (IL-4) and inflammatory signaling (TNF-α), demonstrating their unique advantages in multi-target synergy and multi-level intervention within the immune-inflammatory network, and providing a new paradigm for natural drug intervention in the precise treatment of allergic diseases.

In allergic diseases, MAPK, as a core signaling hub, is closely related to the pathological development of allergy and plays a key role in the initiation, amplification, and chronicity of allergic reactions by regulating immune cell activation, cytokine networks, and the release of inflammatory mediators [33].

In the present study, we investigated the expression levels of key proteins within the MAPK signaling pathway. Protein expression was significantly upregulated in the model group, whereas treatment with drug-containing serum prepared from GRRPM-CDs led to a marked reduction in the activation of this pathway. Treatment with GRRPM-CDs significantly downregulates the phosphorylation levels of key proteins in the MAPK pathway—including p-ERK, p-JNK, and p-p38—in C48/80-induced RBL-2H3 cells, thereby reducing the release of downstream pro-inflammatory cytokines and degranulation mediators. These findings indicate that GRRPM-CDs may exert anti-allergic effects by suppressing the MAPK pathway and thereby attenuating allergic responses.

Although this study initially demonstrated the anti-allergic potential of GRRPM-CDs, several limitations remain that require further clarification.

First, the biological findings of this study are based solely on the in vitro model using the RBL-2H3 mast cell line. There is a lack of validation in more complex in vivo animal models or primary human cells, which limits the direct clinical translatability of the conclusions. The therapeutic potential of GRRPM-CDs requires further confirmation in physiologically more relevant systems. As an exploratory study, the relatively small sample size in the cell-based experiments and the lack of comparison with positive controls—such as standard antihistamines—also limit the assessment of the relative efficacy of GRRPM-CDs. Currently, we are focusing on characterizing the basic properties of the carbon dots, including their morphology, crystalline structure, and surface functional groups. However, many aspects of their characterization remain to be explored. Finally, the observed reduction in cell viability at higher concentrations should be interpreted with caution. Although this suggests a potential therapeutic window, the compound’s safety profile still requires evaluation in vivo.

Despite the aforementioned limitations, this study—as a fundamental exploration—has uncovered the research value of GRRPM-CDs as a novel nanoscale component for anti-allergic applications and has established a methodological foundation for subsequent research.

## 5. Conclusions

GRRPM-CDs are nanocomponents formed during the processing of *Glycyrrhizae Radix et Rhizoma Praeparata cum Melle*. This study demonstrates the significant therapeutic potential of GRRPM-CDs in allergic diseases, as evidenced by in vitro cellular models, and explores their underlying molecular mechanisms. These findings provide novel insights into the active constituents of *Glycyrrhizae Radix et Rhizoma Praeparata cum Melle* involved in the modulation of allergic responses, thereby offering a new perspective on the scientific basis of traditional Chinese herbal processing and supporting the development of innovative anti-allergic therapeutics.

## Figures and Tables

**Table 1:** Effects of different volume fractions of drug-containing serum on RBL-2H3 viability.

Group	24 h Cell Survival Rate (%)	48 h Cell Survival Rate (%)
Control	100 ± 0.49	100 ± 0.59
5% BS	99.19 ± 0.46	98.27 ± 0.17
10% BS	99.68 ± 0.40	98.31 ± 0.18
15% BS	78.50 ± 0.35 ##	53.81 ± 0.55 ##
20% BS	41.85 ± 0.72 ##	26.57 ± 0.53 ##
5% GRRPM-CDs	99.58 ± 0.39	98.39 ± 0.08
10% GRRPM-CDs	99.77 ± 0.48	98.31 ± 0.04
15% GRRPM-CDs	69.50 ± 0.94 ##	62.79 ± 0.98 ##
20% GRRPM-CDs	56.98 ± 0.39 ##	28.57 ± 0.47 ##

Mean ± SD, *n* = 4. Compared to Control, ## *p* < 0.01.

**Table 2:** Effects on β-Hex release in RBL-2H3 cells.

Group	β-Hex Release Amount (ng/mL)
Control	0.61 ± 0.14
C48/80	7.55 ± 0.57 ###
5% BS	6.99 ± 0.56
10% BS	7.29 ± 0.43
5% GRRPM-CDs	5.65 ± 0.29 ###
10% GRRPM-CDs	2.47 ± 0.30 ###

Mean ± SD, *n* = 4. Compared to Control, ### *p* < 0.001.

## Data Availability

The original contributions presented in this study are included in the article. Further inquiries can be directed to the corresponding authors.

## Abbreviations

The following abbreviations are used in this manuscript:

β-Hex	β-hexosaminidase
C48/80	Compound 48/80
CCK-8	Cell Counting Kit-8
ELISA	Enzyme-Linked Immunosorbent Assay
ERK	Extracellular Signal-Regulated Kinase
FL	Fluorescence spectra
FTIR	Fourier-transform infrared
GRRPM	*Glycyrrhizae Radix et Rhizoma Praeparata cum Melle*
GRRPM-CDs	*Glycyrrhizae Radix et Rhizoma Praeparata cum Melle* Carbon Dots
HIS	Histamine
HPLC	High-Performance Liquid Chromatography
HRQL	health-related quality of life
HRTEM	High-resolution transmission electron microscopy
IL-4	Interleukin-4
JNK	c-Jun N-terminal Kinase
MAPK	Mitogen-Activated Protein Kinase
OD	Optical Density
P38	p38 Mitogen-Activated Protein Kinase
RBL-2H3	rat basophil leukemia cells
TEM	Transmission electron microscopy
TNF-α	Tumor Necrosis Factor-α
UV-vis	Ultraviolet–visible absorption spectra
WB	Western blot
XPS	X-ray photoelectron spectroscopy
XRD	X-ray Diffractometer
